# Supplementary material for: Polygenic risk scores for asthma and allergic disease associate with COVID-19 severity in 9/11 responders
Source: PLoS One. 2023 Mar 9;18(3):e0282271. doi: 10.1371/journal.pone.0282271 (PMC9997960; doi:10.1371/journal.pone.0282271)
Supplement: S1 Table — (DOCX) [file pone.0282271.s001.docx]

**Supplementary Materials**

Waszczuk, M. A., Morozova, O., Lhuillier, E., Docherty, A. R. Shabalin, A. A., … Benjamin J. Luft (in sub). Polygenic Risk Scores for Asthma and Allergic Disease Associate with COVID-19 Severity in 9/11 Responders.

Supplementary Table 1 –Associations among PRS used in the study

|  | PRS: Asthma | PRS: Allergic disease | PRS: Coronary artery disease | PRS: Type II diabetes | PRS: COVID-19 hospitalized vs. controls | PRS: COVID-19 hospitalized vs. not-hospitalized |
| --- | --- | --- | --- | --- | --- | --- |
| PRS: Asthma | 1 | .42* | .02 | .04 | .02 | -.01 |
| PRS: Allergic disease | .43* | 1 | -.03 | -.01 | .02 | .00 |
| PRS: Coronary artery disease | .02 | -.02 | 1 | .16* | .09* | .10* |
| PRS: Type II diabetes | .04 | -.01 | .16* | 1 | .12* | .03 |
| PRS: COVID-19 hospitalized vs. controls | .02 | .03 | .06 | .10* | 1 | .63* |
| PRS: COVID-19 hospitalized vs. not-hospitalized | .01 | .01 | .09* | .02 | .65* | 1 |

*Note:* Association in full sample reported below diagonal, associations in European ancestry reported above diagonal. Partial correlations adjusted for the first ten principal components of the population structure are reported.

* indicates *p*<.05
