# Supplementary material for: Polygenic risk scores for asthma and allergic disease associate with COVID-19 severity in 9/11 responders
Source: PLoS One. 2023 Mar 9;18(3):e0282271. doi: 10.1371/journal.pone.0282271 (PMC9997960; doi:10.1371/journal.pone.0282271)
Supplement: S2 Table — (DOCX) [file pone.0282271.s002.docx]

**Supplementary Materials**

Waszczuk, M. A., Morozova, O., Lhuillier, E., Docherty, A. R. Shabalin, A. A., … Benjamin J. Luft (in sub). Polygenic Risk Scores for Asthma and Allergic Disease Associate with COVID-19 Severity in 9/11 Responders.

Supplementary Table 2 – Descriptive statistics for participants of all ancestries

| N total | 983 |
| --- | --- |
| N male (%) | 918 (93.4%) |
| Mean age at infection (SD), range | 56.03 (7.37), 39.79-89.04 |
| N OAD diagnosis (%) | 372 (37.8%) |
| N URD diagnosis (%) | 627 (63.8%) |
| N PTSD and/or MDD diagnosis (%) | 146 (14.9%) |
| Mean BMI (SD), range | 31.31 (5.09), 19.48-62.18 |
| N COVID-19 severity (%) |  |
| Asymptomatic | 92 (9.4%) |
| Mild | 378 (38.5%) |
| Moderate | 408 (41.5%) |
| Severe | 75 (7.6%) |
| Missing | 30 (3.1%) |
| N any residual symptoms | 306 (31.1%) |

*Notes:*

OAD – Obstructive airway disease; URD – upper respiratory disease; BMI – body mass index; COVID-19 – coronavirus disease 2019; SD - standard deviation.
