# Supplementary material for: Polygenic risk scores for asthma and allergic disease associate with COVID-19 severity in 9/11 responders
Source: PLoS One. 2023 Mar 9;18(3):e0282271. doi: 10.1371/journal.pone.0282271 (PMC9997960; doi:10.1371/journal.pone.0282271)
Supplement: S3 Table — (DOCX) [file pone.0282271.s003.docx]

**Supplementary Materials**

Waszczuk, M. A., Morozova, O., Lhuillier, E., Docherty, A. R. Shabalin, A. A., … Benjamin J. Luft (in sub). Polygenic Risk Scores for Asthma and Allergic Disease Associate with COVID-19 Severity in 9/11 Responders.

Supplementary Table 3– Associations between allergic disease and asthma PRS and COVID-19 severity and residual symptoms in participants of all ancestries.

|  | COVID-19 severity | COVID-19 severe category | Any residual symptoms |
| --- | --- | --- | --- |
| PRS: Asthma | ***β*=.08, *p*=.01** | ***OR*=1.53 (CI:1.15-2.03), *p*<.01** | *OR*=1.11 (CI:.95-1.29), *p*=.20 |
| PRS: Allergic disease | *β*=.09, *p*=.05 | ***OR*=1.86**  **(CI: 1.25-2.77), *p*<.01** | *OR*=.94  (CI: .75-1.18), *p*=.59 |

*Notes:*

OR: Odds ratio; CI: 95% confidence interval; PRS: polygenic risk score; COVID-19: coronavirus disease 2019. All models are adjusted for the first ten principal components of the population structure, verification status, age at infection, sex, obstructive airway disease diagnosis, upper respiratory disease diagnosis. Models with residual symptoms as a dependent variable were additionally adjusted for COVID-19 severity.
