# Supplementary material for: Polygenic risk scores for asthma and allergic disease associate with COVID-19 severity in 9/11 responders
Source: PLoS One. 2023 Mar 9;18(3):e0282271. doi: 10.1371/journal.pone.0282271 (PMC9997960; doi:10.1371/journal.pone.0282271)
Supplement: S4 Table — (DOCX) [file pone.0282271.s004.docx]

**Supplementary Materials**

Waszczuk, M. A., Morozova, O., Lhuillier, E., Docherty, A. R. Shabalin, A. A., … Benjamin J. Luft (in sub). Polygenic Risk Scores for Asthma and Allergic Disease Associate with COVID-19 Severity in 9/11 Responders.

Supplementary Table 4 – Associations between coronary artery disease and type II diabetes PRS and COVID-19 severity and residual symptoms in participants of all ancestries.

|  | COVID-19 severity | COVID-19 severe category | Any residual symptoms |
| --- | --- | --- | --- |
| PRS: Coronary artery disease | *β*=.05, *p*=.24 | *OR*=1.11 (CI:.77-1.61), *p*=.57 | *OR*=1.19 (CI:.96-1.47), *p*=.12 |
| PRS: Type II diabetes | *β*=.07, *p*=.27 | *OR*=1.42  (CI: .84-2.40), *p*=.19 | *OR*=.72  (CI: .53-1.00), *p*=.05 |

*Notes:*

OR: Odds ratio; CI: 95% confidence interval; PRS: polygenic risk score; COVID-19: coronavirus disease 2019. All models are adjusted for the first ten principal components of the population structure, verification status, age at infection, sex, and BMI. Models with residual symptoms as a dependent variable were additionally adjusted for COVID-19 severity.
