# Supplementary material for: Polygenic risk scores for asthma and allergic disease associate with COVID-19 severity in 9/11 responders
Source: PLoS One. 2023 Mar 9;18(3):e0282271. doi: 10.1371/journal.pone.0282271 (PMC9997960; doi:10.1371/journal.pone.0282271)
Supplement: S5 Table — (DOCX) [file pone.0282271.s005.docx]

**Supplementary Materials**

Waszczuk, M. A., Morozova, O., Lhuillier, E., Docherty, A. R. Shabalin, A. A., … Benjamin J. Luft (in sub). Polygenic Risk Scores for Asthma and Allergic Disease Associate with COVID-19 Severity in 9/11 Responders.

Supplementary Table 5 – Associations between COVID-19 PRS and COVID-19 severity and residual symptoms in participants of all ancestries.

|  | COVID-19 severity | COVID-19 severe category | Any residual symptoms |
| --- | --- | --- | --- |
| PRS: COVID-19 hospitalized vs. controls | *β*=.04, *p*=.22 | ***OR*=1.42 (CI:1.11-1.82), *p*=.01** | *OR*=1.08  (CI: .92-1.26), *p*=.36 |
| PRS: COVID-19 hospitalized vs. not-hospitalized | *β*=.05, *p*=.15 | ***OR*=1.33 (CI:1.04-1.69), *p*=.02** | *OR*=1.05 (CI:.89-1.24), *p*=.55 |

*Notes:*

OR: Odds ratio; CI: 95% confidence interval; PRS: polygenic risk score; COVID-19: coronavirus disease 2019. All models are adjusted for the first ten principal components of the population structure, verification status, age at 9/11, and sex. Models with residual symptoms as a dependent variable were additionally adjusted for COVID-19 severity.
