# Supplementary material for: Polygenic risk scores for asthma and allergic disease associate with COVID-19 severity in 9/11 responders
Source: PLoS One. 2023 Mar 9;18(3):e0282271. doi: 10.1371/journal.pone.0282271 (PMC9997960; doi:10.1371/journal.pone.0282271)
Supplement: S6 Table — (DOCX) [file pone.0282271.s006.docx]

**Supplementary Materials**

Waszczuk, M. A., Morozova, O., Lhuillier, E., Docherty, A. R. Shabalin, A. A., … Benjamin J. Luft (in sub). Polygenic Risk Scores for Asthma and Allergic Disease Associate with COVID-19 Severity in 9/11 Responders.

Supplementary Table 6 –Mental health and associations between COVID-19 outcomes and PRS for asthma in European ancestry participants

|  | COVID-19 severity | COVID-19 severe category | Any residual symptoms |
| --- | --- | --- | --- |
| Model 1, Asthma PRS  Mental health excluded | ***β*=.09, *p*=.02** | ***OR*=1.50**  **(CI: 1.06-2.12), *p*=.02** | *OR*=1.10  (CI: .91-1.32), *p*=.33 |
| Model 2, Asthma PRS  Mental health adjusted | ***β*=.09, *p*=.03** | ***OR*=1.60**  **(CI: 1.16-2.20), *p*<.01** | *OR*=1.13  (CI: .97-1.32), *p*=.13 |

*Notes:*

OR: Odds ratio; CI: 95% confidence interval; PRS: polygenic risk score; COVID-19: coronavirus disease 2019. All models are adjusted for the first ten principal components of the population structure, verification status, age at infection, sex, obstructive airway disease diagnosis, upper respiratory disease diagnosis. Models for residual symptoms were additionally adjusted for COVID-19 severity.

Model 1 - Subsample without PTSD and/or MDD diagnostic history (N=124 excluded).

Model 2 - Total sample, additional adjustment for PTSD and/or MDD diagnostic history.
